# Infection of Human Dental Pulp Stromal Cells by *Streptococcus mutans*: Shedding Light on Bacteria Pathogenicity and Pulp Inflammation

**DOI:** 10.3389/fcell.2020.00785

**Published:** 2020-08-31

**Authors:** Elodie Maisonneuve, Julie Chevrier, Marie Dubus, Jennifer Varin, Johan Sergheraert, Sophie C. Gangloff, Fany Reffuveille, Cédric Mauprivez, Halima Kerdjoudj

**Affiliations:** ^1^Université de Reims Champagne Ardenne, EA 4691, Biomatériaux et Inflammation en Site Osseux (BIOS), Reims, France; ^2^Université de Reims Champagne Ardenne, UFR d’Odontologie, Reims, France; ^3^Pôle Médecine bucco-dentaire, Hôpital Maison Blanche, Centre Hospitalier Universitaire de Reims, Reims, France; ^4^Université de Reims Champagne Ardenne, UFR de Pharmacie, Reims, France

**Keywords:** dental pulp derived stromal cells, inflammation, internalization, *S. mutans*, pathogenicity

## Abstract

Cariogenic *Streptococcus mutans* (*S. mutans*) is implicated in the dental pulp necrosis but also in cardiovascular tissue infections. Herein, the purpose was to elucidate how human dental pulp derived stromal cells (DPSCs) react toward a direct interaction with *S. mutans*. DPSCs were challenged with *S. mutans*. Following 3 h of interaction, DPSCs were able to internalize *S. mutans* (rate < 1%), and F-actin fibers played a significant role in this process. *S. mutans* persisted in the DPSCs for 48 h without causing a cytotoxic effect. *S. mutans* was, however, able to get out of the DPSCs cytoplasm and to proliferate in the extracellular environment. Yet, we noticed several adaptive responses of bacteria to the extracellular environment such as a modification of the kinetic growth, the increase in biofilm formation on type I collagen and polyester fabrics, as well as a tolerance toward amoxicillin. In response to infection, DPSCs adopted a proinflammatory profile by increasing the secretion of IL-8, lL-1β, and TNF-α, strengthening the establishment of the dental pulp inflammation. Overall, these findings showed a direct impact of *S. mutans* on DPSCs, providing new insights into the potential role of *S. mutans* in infective diseases.

## Graphical Abstract

*S. mutans* and DPSCs interaction.

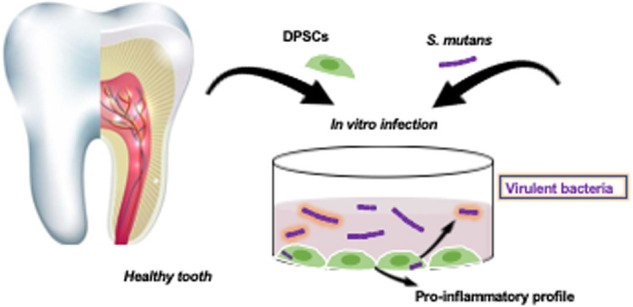

## Introduction

Among tooth colonizers, *Streptococcus mutans* (*S. mutans*) is considered as the primary etiologic agent of dental caries, an infectious disease that affects 60–90% of the population worldwide (Pitts et al., 2017). It is not unusual for *S. mutans* to seek refuge in dentin caries and to gain access to the bloodstream during dental procedures, causing opportunistic systemic infections (Nakano et al., 2006; Carinci et al., 2018). Therefore, bacteremia, through the adhesion to endocardium and collagenous matrix, is responsible amongst others of infective endocarditis and peripheral arterial disease (Abranches et al., 2009; Carinci et al., 2018; Conrads and About, 2018). Dental pulp is a vascularized and innerved connective tissue housed in mineralized dentine. Gronthos et al. isolated and characterized stromal cells from dental pulp tissue (DPSCs) (Gronthos et al., 2009). DPSCs meet minimal core identity for mesenchymal stem cells (MSCs) immunophenotype as defined by International Society for Cellular Therapy (Dominici et al., 2006). As MSCs, DPSCs have pharmaceutical effects that are predominantly mediated by paracrine and contact factors arising from intrinsic MSCs physiological processes (Caplan and Correa, 2011; Wang et al., 2014; Zhou et al., 2020). DPSCs are able to elaborate defense mechanisms against infections with the capacity to participate in cellular defenses and modulate the overall response against microorganisms. Despite of these features, the dental pulp often succumbs to an overwhelming infection. This raises questions about the effect of *S. mutans* on impairing DPSCs defense mechanisms against infections and the underlying systemic infections.

The expression of the immunomodulatory factors in dental derived stromal cells such as DPSCs and dental follicles stem cells is influenced by bacterial products and Toll like receptor (TLR) agonists. Although the expression of the TLR family members in the DPSCs is well documented, their contribution in the immunomodulation is not yet well understood (Andrukhov et al., 2019). It was shown that TLR-3 agonist increases the secretion of TGF-β and IL-6 by both DPSCs and dental follicles stem cells, while TLR-4 agonist increases TGF-β production only by the dental follicles stem cells, but not by DPSCs (Tomic et al., 2010). Stimulation of DPSCs for 48 h with *E. coli*-derived lipopolysaccharide enhances the expression of indolamine-2,3-dioxygenase-1 (Lee et al., 2016), but also increases the expression of pro-inflammatory mediators such as IL-8, IL-1α, and TNF-α in dose dependent manner (Bindal et al., 2018). Despite this knowledge, functional interaction studies of DPSCs and oral pathogenic Gram-positive *S. mutans* and the subsequent inflammatory commitment are lacking.

The focus of our study was to investigate the interactions of human DPSCs with *S. mutans*. We hypothesized that DPSCs were able to internalize *S. mutans*, inducing behavioral changes in bacteria. In response to infection, DPSCs might adopt a proinflammatory profile, contributing to the establishment of the inflammation. These data support the concept that DPSCs serve as a protective niche for *S. mutans* in case of bacterial incursion into the dental pulp. In response, both DPSCs and *S. mutans* adapt their behavior in favor of the establishment of a dental pulp inflammation and bacterial infection.

## Materials and Methods

### DPSCs, Fibroblasts, and *S. mutans* Cultures

Healthy pulp tissues were isolated from non-fully erupted third molars. Following the enzymatic digestion in collagenase and trypsin mixture, the extracted DPSCs were cultured in α-MEM supplemented with 10% decomplemented fetal bovine serum (FBS), 1% Penicillin/Streptomycin/Amphotericin-B and 1% Glutamax^®^ and maintained in a humidified atmosphere of 5% CO_2_ at 37°C. Reaching sub-confluence, DPSCs were seeded at density of 3 × 10^3^ cell/cm^2^ until the third passage. Human gingival derived fibroblasts (HGFs) were isolated from gingival fragments, obtained during teeth removal. HGFs were seeded at density of 5 × 10^3^ cells/cm^2^ in DMEM supplemented with Glutamax^®^ supplemented with 10% FBS and 1% Penicillin/Streptomycin, and maintained in a humidified atmosphere of 5% CO_2_ at 37°C with a medium change every two days. Reaching sub-confluence, HGFs were amplified at density of 10^4^ cell/cm^2^ until the fourth passage. *S. mutans* Clarke-ATCC^®^ 25175 was grown on Columbia agars with 5% sheep blood under anaerobic conditions using GenBag Anaer. For all experiments, *S. mutans* were grown for 24 h in Schaedler broth under aerobic conditions at 37°C.

### Eukaryote Cells/*S. mutans* Interaction

DPSCs and HGFs were seeded in 24-well plates at 10^4^ cells/cm^2^ and cultured in their corresponding medium. After 72 h of culture, cells were washed with DPBS and cultured, overnight, with antibiotic free culture medium. The next day, cells were washed twice with DPBS and 1 mL of antibiotic free culture medium was added. Following enumeration, DPSCS or HGFs were exposed to live *S. mutans* with a multiplicity of infection (MOI) of 30 bacteria: 1 cell. After 3 h of interaction, cells were washed with DPBS and incubated for 1 h with culture medium supplemented by 100 μg/mL of amoxicillin (Panpharma) following antibiotic protection assay procedure. The count of intracellular and extracellular bacteria was determined. At the same time, infected DPSCs were maintained in culture for 24 and 48 h in 1 mL of antibiotic free culture medium. After that, conditioned media (CM) were divided: some parts were filtered and conserved at –80°C, and the rest were plated on blood agar to enumerate the extracellular bacteria.

### Cytochalasin-D Assay

DPSCs were seeded in 24-well plates at 10^4^ cells/cm^2^ and cultured for 72 h. Cells were washed with DPBS and cultured, overnight, in antibiotic free culture medium supplemented with 0.1 μg/mL cytochalasin D (Sigma-Aldrich). Cytochalasin treated cells were incubated with *S. mutans* with a MOI of 30: 1. Following the antibiotic protection assay, the rate of the intracellular bacteria was determined.

### Intracellular Bacteria Quantification

Following 3 h of cell/bacteria interaction and 24 and 48 h culture post-infection (± cytochalasin D treatment), amoxicillin protection assay was performed and cells were lysed with 0.1% Triton X-100 for 5 min. Cell lysates were plated on blood agar and cultured for 24 h under anaerobic conditions, at 37°C. The rate of viable intracellular *S. mutans* (i-*S. mutans*) was determined after bacterial count on agar plate.

### Extracellular Bacteria Quantification

Following 3 h of cell/bacteria interaction and 24 and 48 h culture post-infection (± cytochalasin D treatment), the harvested conditioned media were plated on blood agar and cultured for 24 h under anaerobic conditions, at 37°C. The rate of viable extracellular *S. mutans* (e-*S. mutans*) was determined after bacterial count on agar plate.

### Growth Monitoring

The bacteria growth was monitored for 72 h. Each isolated colony was added in 6 mL of Schaedler broth. The optical density (OD) of bacteria suspension was measured at 600 nm in a spectrophometer cuve. Curves were achieved by calculating ln(N/N_0_) (N = OD at the analyzed time; N_0_ = starting OD) (Zwietering et al., 1990).

### Minimum Inhibitory Concentration (MIC) and Minimum Bactericidal Concentration (MBC)

Amoxicillin (100–0.10 μg/mL range) and vancomycin (25–0.10 μg/mL range) in Schaedler-broth were used. Bacteria (10^6^ bacteria/mL) were added and incubated at 37°C for 24 h. MIC values were determined as the lowest concentrations of antibiotic having an inhibitory effect on bacterial growth. After 24 h of incubation, the lowest concentration of the subculture (10 μL) showing no growth on blood agar was considered as the MBC.

### *S. mutans* Adhesion and Biofilm Formation on Type I Collagen (COL-I)

24-well plates were coated with 100 μg/mL of COL-I and incubated with 500 μL of bacteria suspension (10^6^ bacteria/mL) at 37°C. After 6 h of culture, adhered bacteria were exposed to ultrasound (40 kHz) for 5 min and a volume of 100 μL from serial dilutions was plated on blood agars. After 24 h of culture the plates were gently washed and 2 mL of 0.2% crystal violet was added to determine the quantity of attached bacteria. After 20 min of incubation, unfixed crystal violet was removed and absolute ethanol was added. OD was measured at 595 nm. Controls were carried out without bacteria.

### *S. mutans* Adhesion to Polyester Fabrics and Scanning Electron Microscopy

The polyester braided fabrics, based on multifilament yarn, are typically used in cardiovascular applications (provided by CRITT-TJFU). Fabrics were incubated for 6 h with bacteria suspension (10^6^ bacteria/mL) at 37°C. Attached bacteria were determined as described above. Samples were fixed in 2.5% glutaraldehyde for 1 h, dehydrated in graded ethanol solutions for 10 min and desiccated in a drop of hexamethyldisilazane. After air-drying at room temperature, samples were sputtered with a thin gold-palladium film. Samples were observed using Scanning Electron Microscope (SEM-JEOL-JSM-7900F).

### Lactate Dehydrogenase (LDH) Cytotoxicity Assay and DNA Quantification

LDH activity was evaluated in conditioned media of dental pulp derived stromal cells (DPSCs-CM) with the cytotoxicity detection kit following the manufacturer’s instructions. OD was measured at 492 and 700 nm. DNA extraction was performed on DPSCs, 48 h post-infection using MasterPure^TM^ DNA Purification Kit in accordance with the manufacturer’s protocol. Extracted DNA was assessed by measuring the absorbance at 260 and 280 nm.

### Fluorescence Labeling

Before challenging, *S. mutans* were labeled using 4′,6-diaminido-2-phenylindole (DAPI) and incubated for 10 min at 37°C. After 3 h of interaction, DPSCs were fixed with 4% paraformaldehyde at 37°C for 15 min and permeabilized with 0.5% Triton X-100 for 10 min. DPSCs were then incubated for 30 min at room temperature with Alexa^®^ Fluor-488-conjugated-Phalloidin^®^. Stained cells were mounted and imaged by confocal microscopy (LSM 710, NLO, Zeiss, Germany).

### Cytokine Release by ELISA

DPSCs-CM were analyzed. Secreted levels of IL-8, TNF-α, and IL-1β were assessed using ELISA MAX^TM^ Deluxe Set Human IL-8 and ELISA MAX^TM^ Deluxe Set Human TNF-α and Duoset^®^ human IL-1β, respectively, according to the manufacturer’s instructions. OD were measured at 450 nm using a microplate reader.

### Statistical Analysis

All statistical analyses were performed using GraphPad Prism 6 software. DPSCs experiments were performed with six-independent healthy donors. HGFs experiments were performed with three-independent healthy donors. Microbiological assays were performed at least three times in triplicates. Histograms represent mean ± SEM and statistical analyses were performed using Mann & Whitney test. For each test, a value of *p* < 0.05 was accepted as statistically significant.

## Results and Discussion

**FIGURE 1:**
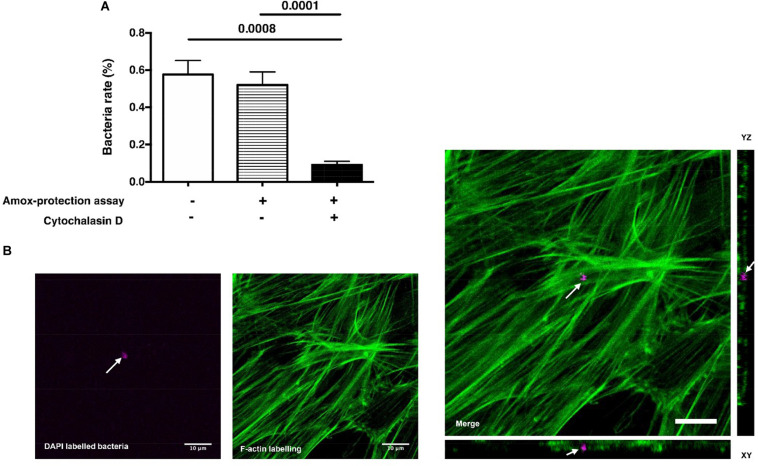
*S. mutans* internalization. **(A)** Percentage of viable *S. mutan*s after 3 h of contact with dental pulp stem cells (DPSCs). Tests were performed with or without amoxicillin treatment (Amox-protection assay). *S. mutans* challenged with Cytochalasin D-treated DPSCs indicates that F-actin fibers are required for *S. mutans* internalization by DPSCs (Histograms of mean ± SEM, *n* = 6, Mann & Whitney test). **(B)** Laser scanning confocal microscopy visualization and orthogonal view of DPSCs infected with *S. mutans*. Green color corresponds to cytoskeleton labeled with Phalloidin-coupled to Alexa^®^ 488 and magenta color corresponds to *S. mutans* labeled with DAPI. White arrows indicate bacteria. Scale bar = 10 μm.

Following a deep carious lesion, the dental pulp tissue establishes efficient strategies to hinder or even arrest the carious lesion progression and the bacterial incursion into the pulp (Conrads and About, 2018). In this study, DPSCs did not exert a direct antibacterial effect on *S. mutans*; instead, bacteria seem to have great ability to thrive under DPSCs environmental conditions (Supplementary Figure SI-1; LaRock and Nizet, 2015). *S. mutans* was found to adhere to the eukaryote cell membranes but without being invasive (Engels-Deutsch et al., 2003; Abranches et al., 2009; Berlutti et al., 2010; Kaufman and Skrtic, 2016). In following, we investigated if *S. mutans* adhere to DPSCs and if the DPSCs might serve as a protective niche for *S. mutans* in case of bacterial incursion into the dental pulp. The rate of viable *S. mutans* showed that, following 3 h of contact, bacteria were able to invade DPSCs without affecting the cell integrity (Figure 1). To clarify the relationship between bacterial attachment and bacterial invasion, colony forming unit (CFU) counts were performed following extensive rinse with PBS or following amoxicillin-based antibiotic protection assay (Figure 1A). *S. mutans* is sensitive to amoxicillin and the addition of this antibiotic is expected to kill the extracellular bacteria but not the intracellular bacteria (Scaglione et al., 1993). The rate of the viable bacteria was < 1%, with very close CFU number with or without antibiotic protection assay (Supplementary Figure SI-2). These results suggest a neglecting number of adhered bacteria to DPSCs or a weak adhesion that was broken during washing procedure. Further quantification of bacterial adhesion forces to DPSCs should be conducted by using atomic force microscopy. Interestingly, HGFs were not able to prone to *S. mutans* adhesion and invasion. These results are in contradiction with (Berlutti et al., 2010) who demonstrated that 0.3% of *S. mutans* (ATCC^®^ 25175) are able to adhere and invade gingival fibroblast cell line. To confirm the invasion ability of *S. mutans* into DPSCs, DAPI labeled bacteria were used. DPSCs cytoskeleton was labeled and infected cells were observed by confocal microscopy. Infected DPSCs showed the presence of polymerized F-actin, signature of the preservation of the integrity of the adhered cells. The cross-sectional analysis of stacked confocal images revealed the presence of bacteria setting between F-actin, confirming cytoplasm invasion (Figure 1B). The plasma membrane of eukaryotic cells is a dynamic structure that coordinates the entry of macromolecules and cells by endocytic pathways (Gruenberg and van der Goot, 2006). To examine the role of F-actin and microtubules in *S. mutans* entry, DPSCs were treated with cytochalasin-D (i.e., known to cause microfilament depolymerization) for 24 h prior bacteria challenging. Compared to untreated DPSCs, the results showed that cytochalasin-D inhibited *S. mutans* uptake by ∼ 82%, suggesting that F-actin is critical for *S. mutans* internalization (Figure 1A). To summarize, *S. mutans* are able to invade DPSCs. Despite a not detectable adhesion of *S. mutans* to DPSCs plasma membrane, F-actin fibers are required for *S. mutans* internalization.

**FIGURE 2:**
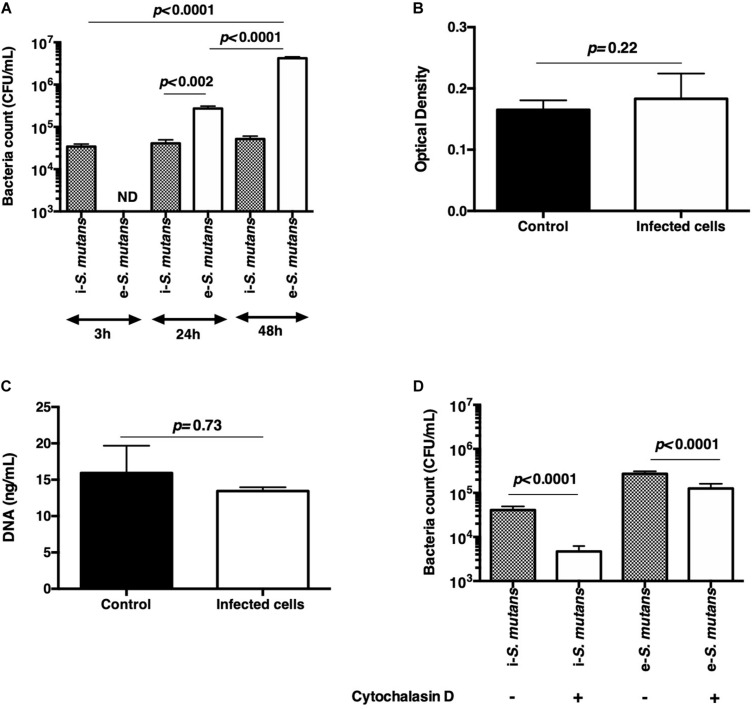
Bacteria persistence. **(A)** Count of viable intracellular (i-*S. mutans)* and extracellular (e-*S. mutans)* bacteria at 3, 24, and 48 h after internalization. Results indicate the absence of intracellular proliferation and the release of *S. mutans* in the extracellular environment. **(B,C)** Lactate dehydrogenase cytotoxicity assay and DNA quantification of DPSCs, respectively, indicating the absence of bacteria cytotoxicity after 48 h of culture. **(D)** Count of viable intracellular and extracellular *S. mutans*, after 24 h of culture of infected DPSCs in the absence and the presence of cytochalasin D. (Histograms of mean ± SEM, *n* = 6; Mann & Whitney test; ND = not detected).

Some pathogens could persist and proliferate in eukaryotic cells without affecting their integrity (Pizarro-Cerdá and Cossart, 2006). The intracellular persistence and proliferation of *S. mutans* was investigated by treating infected DPSCs for 1 h with amoxicillin and then leaving the DPSCs in antibiotic-free media for 24 and 48 h. *S. mutans* were able to survive in the DPSCs over the study time (Figure 2A). The CFU of viable intracellular (i)-*S. mutans* was similar to those detected after 3 h of interaction. Interestingly, viable extracellular bacteria (e)-*S. mutans* were detected after 24 and 48 h of internalization. The recovery rates of e-*S. mutans* after 24 and 48 h were about 700% and 10,500% of the internalized *S. mutans*, respectively. Taken together, these results suggest that *S. mutans* are able to get out of cytoplasm and to proliferate in the extracellular environment. The most likely hypotheses are the death of infected DPSCs causing the bacteria release, or the externalization of *S. mutans* by infected DPSCs. It has been reported that lipoteichoic acid has a cytotoxic and apoptotic effect on DPSCs (Wang et al., 2001), while other reported an increase in the size of microtissue iMDP-3 and proliferation of the fibroblast following challenging with *S. mutans in vitro* (Kaufman and Skrtic, 2016; Nomura et al., 2016). Fourty eight hours post-infection, lactate dehydrogenase (LDH) accumulation in DPSCs-CM was measured and DNA of adhered DPSCs was quantified. LDH is a stable cytoplasmic enzyme present in most cells. During damage to cell cytoplasmic membranes, LDH is released from cells and into the surrounding cell-culture supernatant. Thus, the quantitation of LDH in cell-culture supernatant is one method by which investigators analyze cell-death levels. Compared to uninfected DPSCs, no significant differences in LDH accumulation and in DNA content were noticed (Figures 2B,C), suggesting a negligible DPSCs lysis following *S. mutans* internalization but also the absence of DPSCs proliferation. Thus, the absence of cytotoxic effect is in accordance with (Kaufman and Skrtic, 2016; Nomura et al., 2016) results. We examined whether cytoskeleton compounds are implicated in the bacteria release. By culturing infected DPSCs for 24 h in the presence of cytochalasin-D, viable e-*S. mutans* were detected, suggesting that cytoskeleton does not play a role in bacteria externalization (Figure 2D). However, the rates of both i-*S. mutans* and e-*S. mutans* were lower than control (DPSCs in the absence of cytochalasin-D).

**FIGURE 3:**
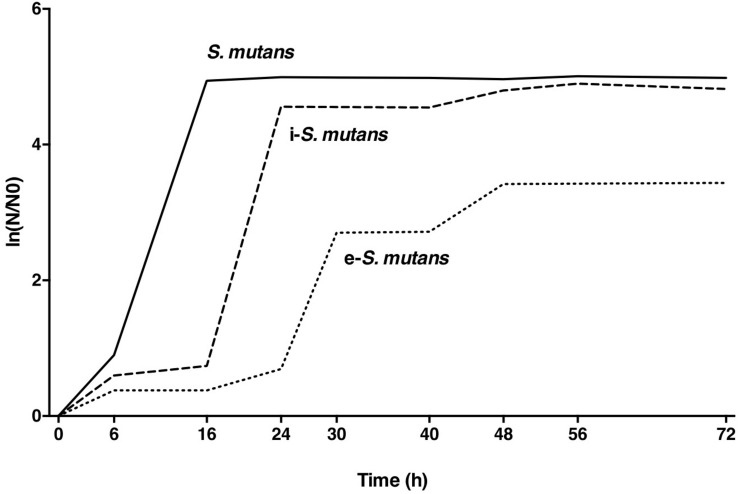
Representative growth curves of the intracellular (i-*S. mutans*), the extracellular (*e-S. mutans*) and the bacteria control (*S. mutans)*, indicating a biphasic growth for *e-S. mutans* (*n* = 3).

The pathogenicity and the virulence of both i-*S. mutans* and e-*S. mutans* isolated after 48 h post-infection was investigated and compared to the non-internalized bacteria. First, the growth curves were performed by transferring the harvested bacteria from the blood agar into fresh growth medium. In contrast to i-*S. mutans* and bacteria control that exhibited a monophasic growth pattern, e- *S. mutans* showed a biphasic growth pattern. Compared to bacteria control, i-*S. mutans* and e-*S. mutans* showed a longer lag phase (respectively, 16 and 24 h versus 6 h; Figure 3), indicating a delay in the bacteria growth following the contact with DPSCs. This could correspond to a period of adjustment that is needed for the establishment of the regulation of metabolic genes responsible for bacteria proliferation (LaRock and Nizet, 2015). Carbohydrates are common carbon and energy sources for all Streptococci. With certain pairs of sugar, a monophasic growth pattern was observed, whereas, others gave a biphasic growth pattern (i.e., diauxic growth) (Janda and Kuramitsu, 1978; Narang and Pilyugin, 2007). The diauxic lag reflects the time required for bacteria to build up the enzymes to sufficiently high levels, for the consumption of preferentially carbohydrate sources. Thus, the prolonged contact of *S. mutans* with DPSCs might affect enzyme synthesis and/or activity required for the carbohydrate catabolism.

Amoxicillin and vancomycin are effective antimicrobials against *S. mutans* and are commonly used in the antibiotic prophylaxis and/or in the antibiotic therapy of infective endocarditis (Habib et al., 2015; Limeres Posse et al., 2016). In 2019, a clinical study estimated the amoxicillin resistance was associated with higher mortality in a large cohort of patients with streptococci-related infective endocarditis (Pilmis et al., 2019). We investigated the susceptibility of e-*S. mutans*, i-*S. mutans* and bacterial control to amoxicillin and vancomycin, through the determination of MBC and MIC (Table 1). Amoxicillin showed a MBC: MIC ratio < 1 for *S. mutans* control, meanwhile, i-*S. mutans* and e-*S. mutans* exhibited an increase in MBC: MIC ratio (8 and 64, respectively). Vancomycin showed a MBC: MIC ∼ 16 for *S. mutans* and a decrease in MBC: MIC for both i-*S. mutans* and e-*S. mutans* (1 and 8, respectively). When the MBC is close to the MIC, the antibiotic is bactericidal; if MBC: MIC ratio is between 4 and 16, the antibiotic is bacteriostatic; and finally, if MBC: MIC ratio is > 32, we speak of “tolerance” (Brauner et al., 2016; Dafale et al., 2016). We can thus conclude that vancomycin had a bactericidal effect on i-*S. mutans* and bacteriostatic effect on e-*S. mutans*. Amoxicillin had a bacteriostatic effect on i-*S. mutans* while e-*S. mutans* seemed tolerant. This feature seems transitory and lost following the *in vitro* bacteria expansion. Antibiotic tolerance is associated with the failure of antibiotic treatment and the relapse of many bacterial infections (Brauner et al., 2016). The most likely hypothesis is the environmental changes and the metabolic adaptations of bacteria along with the prolonged lag phase of e-*S. mutans* (Brauner et al., 2016). We also cannot exclude the possibility of alteration or over expression of penicillin-binding-proteins (PBPs) in contact with DPSCs. Although the primary mode of action of vancomycin and amoxicillin is the inhibition of cell wall synthesis, their targets are different. Vancomycin binds very tightly to cytoplasmic peptides while amoxicillin binds PBPs (Watanakunakorn, 1984; Macheboeuf et al., 2006). Thus, the slower growth rate and a possible alteration of cell-wall could make e-*S. mutans* less susceptible to amoxicillin.

**TABLE 1:** The Minimal Inhibitory Concentration (MIC) and Minimal Bactericidal Concentration (MBC) in μg/mL of amoxicillin and vancomycin to the intracellular (i-*S. mutans*), the extracellular (e-*S. mutans*), and the bacteria control (*S. mutans*) (*n* = 3).

	Amoxicillin			Vancomycin		
	MIC	MBC	MBC: MIC ratio	MIC	MBC	MBC: MIC ratio
*S. mutans*	<0.10	0.10	< 1	0.78	12.5	16
i-*S. mutans*	0.10	0.78	8	0.39	0.39	1
e-*S. mutans*	0.10	6.25	64	0.39	3.12	8

**FIGURE 4:**
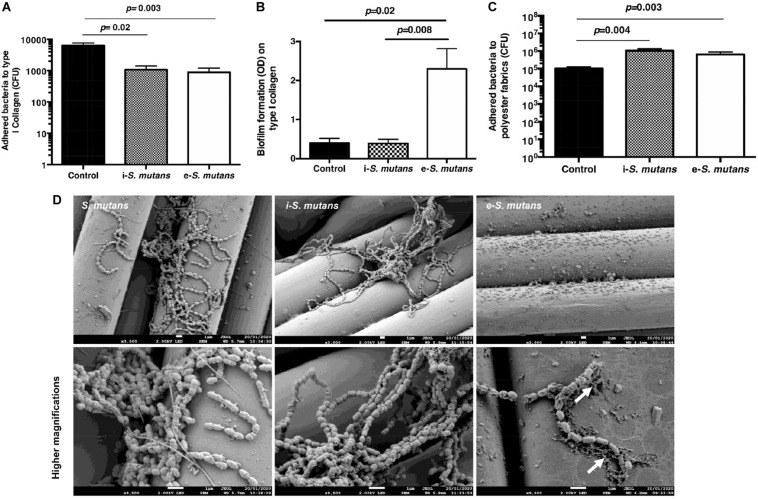
*S. mutans* adhesion and biofilm formation. **(A,B)** Intracellular (i-*S. mutans*), extracellular (e-*S. mutans*), and bacteria control (*S. mutans*) adhesion and biofilm formation on type I collagen coating, respectively. **(C)** Intracellular (i-*S. mutans*), extracellular (e-*S. mutans*) and bacteria control (*S. mutans*) adhesion on polyester fabrics. (Histograms of mean ± SEM, *n* = 4; Mann & Whitney test). **(D)** Scanning electron microscopy visualization of adhered intracellular (i-*S. mutans*), extracellular (e-*S. mutans*) and bacteria control (*S. mutans*) on polyester fabrics. White arrows indicate matrix formation. Lower row indicates a higher magnification of the upper row. Scale bars = 1 μm.

Bacteria can adhere and form biofilms (Lemos et al., 2019). The pathogenesis of *S. mutans* is associated with their adhesion to the extracellular matrix such as collagen and their ability to form biofilms on solid surfaces (i.e., valve implants). We thus analyzed the adhesion of i-*S. mutans* and e-*S. mutans* on either COL-I coating or polyester fabrics (i.e., raw material for valve implant and cardiovascular devices) (Figure 4). Compared to the control, i-*S. mutans* and e-*S. mutans* showed lower adhesion capabilities to COL-I (Figure 4A). Based on biofilm staining, the crystal violet assay highlighted that e-*S. mutans* increased significantly the formation of biofilm on COL-I versus i-*S. mutans* and control (Figure 4B). Bacterial biofilm is a community of adhered bacteria to a surface, which are embedded in a self-produced polymeric substance. Crystal violet is a basic protein dye that stains viable bacteria, dead bacteria, and polymeric substances. The low number of the adhered e-*S. mutans* on COL-I, and the high retention of the crystal violet dye by e-*S. mutans* suggested that these bacteria develop a higher capability to form a matrix *versus* i-*S. mutans* and control. On polyester fabrics, both i-*S. mutans* and e-*S. mutans* showed an increase in adhesion capability compared to control (Figure 4C). Due to high non-specific fixation of crystal violet to the polyester fabrics, the biofilm was not determined. However, SEM pictures seem to indicate a matrix deposition on the polyester yarn by e-*S. mutans* (Figure 4D). Taken together, these results suggest that DPSCs interaction affects the surface motif adhesions of *S. mutans* strains.

**FIGURE 5:**
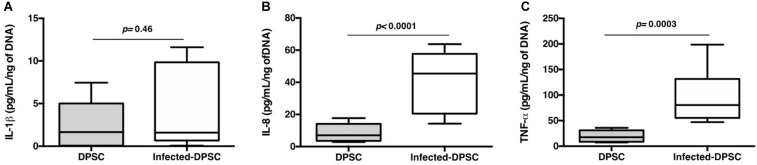
Proinflammatory response. Released IL-1β **(A)**, IL-8 **(B)**, and TNF-α **(C)** detected by ELISA (*n* = 6; Mann & Whitney test).

During a carious disease, the host aims to fight the infection, through its immune-inflammatory response, and to restore the tooth structure *via* the DPSCs commitment. Healthy dental pulp contains resident immune cells such as leukocytes that are able to detect the invaded pathogens and to release plethora of chemoattractive mediators (Farges et al., 2015). Studying the interaction of DPSCs with various components of both innate and adaptive immune systems showed that DPSCs and activated immune cells reciprocally regulate each other. However, DPSCs might adopt either an immunosuppressive or immunostimulatory phenotype depending on the level of inflammation (Andrukhov et al., 2019). The above results established that DPSCs survive to *S. mutans* infection. These data prompted us to explore the presence of pro-inflammatory mediators in the DPSCs-CM. While *S. mutans* had a moderate effect on IL-1β secretion, we observed a significant increase in both IL-8 and TNF-α production following infection (Figure 5). Temporal and contextual profiles of IL-8, TNF-α, and IL-1β might exert negative effects on tissue repair leading to inflammation and pulp necrosis (Zanini et al., 2017). We can conclude that in contact with *S. mutans*, DPSCs get a pro-inflammatory profile, that strengthen the establishment of the dental pulp inflammation.

## Conclusion

This study provides new insights into the interaction between *S. mutans* and DPSCs. DPSCs internalize *S. mutans*, providing a protective niche during dental pulp infection. Internalized *S. mutans* were not able to proliferate into the cytoplasm, but were able to be externalized in the extracellular environment where they proliferated. A prolonged contact with DPSCs metabolites seems to induce adaptative profile and bacteria pathogenicity such as an increasing biofilm formation and an establishment of a tolerance toward amoxicillin. Although the cytotoxic effect of *S. mutans* toward DPSCs was not observed, these findings showed that *S. mutans* affect DPSCs paracrine activities by promoting the secretion of pro-inflammatory mediators. *S. mutans* has a high genetic variation and is classified into four serotypes (Bedoya-Correa et al., 2019). The aforementioned findings used one strain of *S. mutans* (ATCC 25175 strain). Therefore, in perspective, the use of clinical strains of *S. mutans* with various serotypes should be performed to confirm these results. Furthermore, to decipher the relationship between DPSCs and *S. mutans* pathogenicity, a genomic and proteomic analyses should be performed. Finally, the cross-talk between infected DPSCs and immune cells could bring additional insights about the inflammatory profile of DPSCs.

## Data Availability Statement

All datasets generated for this study are included in the article/Supplementary Material, further inquiries can be directed to the corresponding author.

## Author Contributions

EM, JC, MD, JV, and JS contributed to conception, design, data analysis and interpretation, and drafted and critically revised the manuscript. FR, SG, CM, and HK contributed to data acquisition and interpretation and critically revised the manuscript. All authors gave final approval and agreed to be accountable for all aspects of the work.

## Conflict of Interest

The authors declare that the research was conducted in the absence of any commercial or financial relationships that could be construed as a potential conflict of interest.
