# Supplementary material for: Infection of Human Dental Pulp Stromal Cells by Streptococcus mutans: Shedding Light on Bacteria Pathogenicity and Pulp Inflammation
Source: Front Cell Dev Biol. 2020 Aug 31;8:785. doi: 10.3389/fcell.2020.00785 (PMC7487799; doi:10.3389/fcell.2020.00785)
Supplement: Supplementary file 1 [file Data_Sheet_1.pdf]

Supplementary Material

Infection of human dental pulp stromal cells by *Streptococcus mutans*: shedding light on bacteria pathogenicity and pulp inflammation

Elodie Maisonneuve[1], Julie Chevrier[1], Marie Dubus[1,2], Jennifer Varin[1], Johan Sergheraert[1,2,3], Sophie C. Gangloff[1,4], Fany Reffuveille[1,4], Cédric Mauprivez[1,2,3], Halima Kerdjoudj[1,2,*]

1 Université de Reims Champagne Ardenne, EA 4691, Biomatériaux et Inflammation en Site Osseux (BIOS), Reims, France .

2 Université de Reims Champagne Ardenne, UFR d'Odontologie, Reims, France.

3 Pôle Médecine bucco-dentaire, Hôpital Maison Blanche, Centre Hospitalier Universitaire de Reims, France.

4 Université de Reims Champagne Ardenne, UFR de Pharmacie, Reims, France.

* **Correspondence:**

Dr. Halima KERDJOUDJ

halima.kerdjoudj@univ-reims.fr; Tel: (+33) 3 26 91 80 12

Materials and Methods

Eukaryotic cells/ *Streptococcus mutans* interaction and culture: DPSCs and HGFs were seeded in 24-well plates at 10^4 cells/cm^2 and cultured in their corresponding medium. After 72 h of culture, cells were washed with DPBS and cultured, overnight, with antibiotic free culture medium. The next day, cells were washed twice with DPBS and 1 mL of antibiotic free culture medium was added. Cells were exposed to live *S. mutans* with a multiplicity of infection (MOI) of 30 bacteria : 1 cell. During infection, to determine bacteria content in the supernatant, 30 µL of cell culture supernatants were collected each hour (T0, T1, T2 and T3 h). The rate of viable *S. mutans* was determined after bacterial count on agar plate.

Supplementary Data

Herein, we sought to investigate if DPSCs exert a direct antibacterial effect against *S. mutans*. Primary cultured human DPSCs were exposed to live *S. mutans*. HGFs and cell- and antibiotics-free medium were used as controls. Compared to the inoculum, *S. mutans* content increased significantly following the first hour of incubation ($p < 0.01$) in controls (Fig. SI-1). Despite a delay in the increase in *S. mutans* content in contact with DPSCs (*i.e.* starting 2 h, $p<0.02$), these results showed that DPSCs did not exert a direct antibacterial effect on *S. mutans*; instead, bacteria seem to have great ability to thrive under DPSCs environmental conditions (LaRock and Nizet 2015).

Supplementary Figures

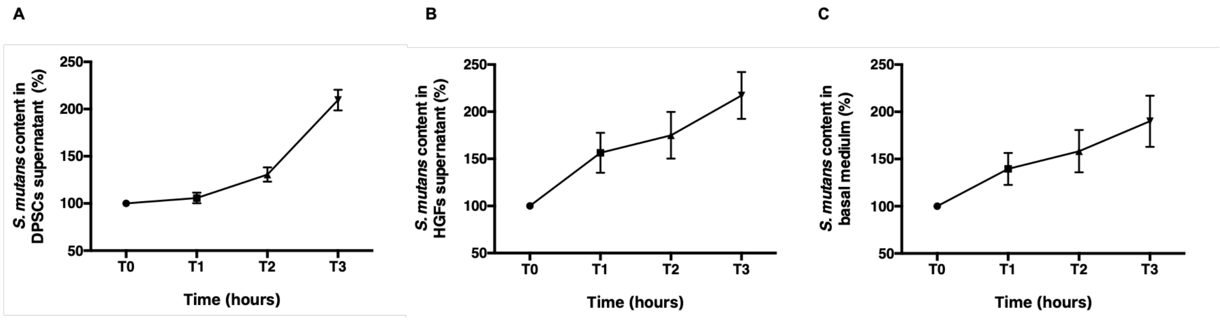

Fig. SI-1: *S. mutans* content in the extracellular environment. During A: dental pulp stromal cells (DPSCs), B: human gingival derived fibroblasts (HGFs) and C: cell free-a-MEM interaction time, suggesting that *S. mutans* established an adaptative behaviour in contact with DPSCs to thrive. Results were normalized to the initial inoculum.

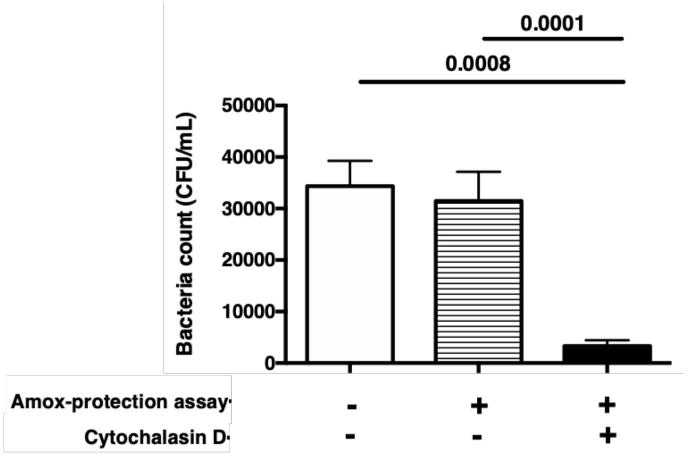

Fig. SI-2: Count of viable *S. mutan*s after 3 h of contact with dental pulp stromal cells (DPSCs). Tests were performed with or without amoxicillin treatment (Amox-protection assay). *S. mutans* challenged with Cytochalasin D-treated DPSCs indicates that F-actin fibres are required for *S. mutans* internalization by DPSCs (Histograms of mean ± SEM, n = 6, Mann & Whitney test).
